# A161 SYMPTOMATIC GASTRIC SCHWANNOMA DIAGNOSED WITH ENDOSCOPIC ULTRASOUND AND FINE NEEDLE BIOPSY: A CASE REPORT

**DOI:** 10.1093/jcag/gwac036.161

**Published:** 2023-03-07

**Authors:** D Wang, C Stallwood

**Affiliations:** McMaster University, Hamilton, Canada

## Abstract

**Background:**

Gastric schwannomas are exceedingly rare neoplasms that account for 0.2% of all gastric masses. They belong to a group of peripheral nerve sheath tumors, which are typically slow-growing, benign lesions that also include neurofibromas and perineuriomas. These neoplasms are often found incidentally on endoscopy as subepithelial lesions.

**Purpose:**

We aim to outline the presentation and differential diagnosis of gastric peripheral nerve sheath tumors.

**Method:**

We present the case of a 44 year old female with heartburn and dyspepsia who was referred for esophagogastroduodenoscopy, which incidentally found a subepithelial lesion on the greater curve of the stomach. She was then referred for EUS-guided fine needle biopsy of the mass, and the pathology was initially reported as a neurofibroma. Eventually, the lesion was surgically removed with the final pathology report stating that this was a gastric Schwannoma. Both specimens had immunostains positive for S-100 and negative for CD34, desmin, DOG1 and cKit.

**Result(s):**

While gastric schwannomas are typically benign, they can still cause abdominal pain, gastrointestinal bleeding, and even gastric outlet obstruction and intussusception. The differential diagnosis of gastric schwannoma includes other peripheral nerve sheath tumors, as well as malignant subepithelial lesions such as gastrointestinal stromal tumors or gastric lymphoma.

**Conclusion(s):**

Gastric schwannoma should always be considered when evaluating subepithelial gastric lesions, and EUS with histologic sampling should be pursued to diagnose and guide the management of these lesions.

**Please acknowledge all funding agencies by checking the applicable boxes below:**

None

**Disclosure of Interest:**

None Declared

